# Valvuloarterial Impedance in End-Stage Kidney Disease Patients With Aortic Stenosis: Echocardiographic Correlates and Outcomes

**DOI:** 10.1016/j.jacadv.2025.101837

**Published:** 2025-05-30

**Authors:** Fredrick M. Ogugua, Lucas Zellmer, Julien Ternacle, Philippe Pibarot, Roy O. Mathew, Gautam R. Shroff

**Affiliations:** a Division of Cardiology, University of Illinois-Chicago, Chicago, Illinois, USA; b Health, Homelessness, and Criminal Justice Lab, Hennepin Healthcare Research Institute, Minneapolis, Minnesota, USA; c Hôpital Cardiologique Haut-Lévêque, CHU de Bordeaux, Pessac, France; d Department of Medicine, Québec Heart and Lung Institute, Laval University, Québec City, Québec, Canada; e Division of Nephrology, Loma Linda VA Medical Center, Loma Linda, California, USA; f Division of Cardiology, Hennepin County Medical Center, University of Minnesota Medical School, Minneapolis, Minnesota, USA

**Keywords:** aortic stenosis, aortic valve replacement, end-stage kidney disease, valvuloarterial impedance

**What is the clinical question being addressed?**

Does elevated Zva predict AVR or mortality in patients with coprevalent and ESKD?

**What is the main finding?**

Zva was not associated with AVR, mortality, or their combined outcome in patients with coprevalent ESKD and AS.

Valvuloarterial impedance (Zva) reflects the global hemodynamic load experienced by the left ventricle (LV) in aortic stenosis (AS). Prior studies have shown that Zva associates with higher risk of mortality among patients with at least moderate asymptomatic AS and predicts unfavorable quality of life/exercise performance following aortic valve replacement (AVR).^1^^,^^2^ Assessment of the global hemodynamic load is especially pertinent in patients with end-stage kidney disease (ESKD), given higher prevalence of hypertension, LV hypertrophy, nonphysiological flow states, and higher risk of adverse outcomes from AS. ^3^ However, prior studies evaluating Zva have excluded patients with ESKD. ^2^^,^^4^ We sought to evaluate the association between Zva, echocardiographic variables and outcomes in patients with coprevalent AS and ESKD.

## Methods

This was a retrospective cohort study of consecutive adults with ESKD receiving dialysis with coprevalent AS from a single safety net health system, Hennepin Healthcare, between January 2000 and March 2021. Patients opting out of research were excluded from the analysis. The study was reviewed by the Institutional Review Board and deemed exempt. AS severity was assessed by transthoracic echocardiography using conventional definitions. Zva (mm Hg/mL/m^2^) was calculated as systolic blood pressure + mean gradient divided by the indexed stroke volume (SVi). The association between Zva and the combined primary outcome of AVR or mortality was assessed using Cox proportional hazard analysis. We also evaluated the association between echocardiographic measures of AS and Zva.

## Results

The cohort included 94 patients with coprevalent ESKD and AS; mean age 66 years, 71% male; and 43% Black. Mild, moderate, and severe AS was observed in 42%, 34%, and 24%, respectively. Among patients with severe AS (N = 22), 18 patients had normal flow-normal gradient severe AS and 2 patients each had low-flow, low-gradient AS and paradoxical low-flow, low-gradient AS. There were no significant differences in demographics, medical history, and etiology of ESKD between patients with median Zva <4 or >4 mm Hg/mL/m^2^ (Table 1). Zva >4 mm Hg/mL/m^2^ was associated with a smaller aortic valve area (AVA) (1.05 vs 1.32 cm^2^; *P* < 0.001), lower SVi (46 vs 33 mL/m^2^; *P* < 0.001), and lower cardiac output (CO) (5.3 vs 6.1 L/min; *P* = 0.005). On univariable analysis, Zva was not associated with the primary composite outcome of AVR and mortality (HR: 0.96; 95% CI: 0.73-1.25; *P* = 0.76). On multivariable analysis, adjusting for demographic factors and aortic valve metrics, Zva was not associated with the primary composite outcome of AVR and mortality (HR: 0.98; 95% CI: 0.75-1.29; *P* = 0.91).

**Table 1:** Comparison of Demographic, Clinical, Echocardiographic Variables, and Outcomes by Valvulo-Arterial Impedance (Zva) Groups

	Zva <4 mm Hg/mL/m^2^ (n = 46)	Zva ≥4 mm Hg/mL/m^2^ (n = 48)	*P* Value
Age, y (mean ± SD)	65.17 (14.44)	67.27 (10.38)	0.42
Race (%)			0.22
Black	18 (39.1)	22 (45.8)	
Other	11 (23.9)	5 (10.4)	
White	17 (37.0)	21 (43.8)	
Male (%)	29 (63.0)	38 (79.2)	0.134
Coronary artery disease (%)	5 (10.9)	4 (8.3)	0.946
Congestive heart failure = yes (%)	5 (10.9)	5 (10.4)	1.00
Atrial fibrillation = yes (%)	3 (6.5)	5 (10.4)	0.759
End-stage kidney disease etiology (%)			0.42
Diabetes	22 (47.8)	19 (39.6)	
Glomerulonephritis	5 (10.9)	7 (14.6)	
Hereditary/cystic	5 (10.9)	4 (8.3)	
Hypertension	6 (13.0)	13 (27.1)	
Other/Unknown	8 (17.4)	5 (10.4)	
Dialysis access types (%)			0.777
Arteriovenous fistula	23 (50.0)	27 (56.2)	
Arteriovenous graft	9 (19.6)	7 (14.6)	
Central venous access	8 (17.4)	6 (12.5)	
Peritoneal dialysis catheter	6 (13.0)	8 (16.7)	
Echocardiographic variables (mean ± SD)			
Systolic blood pressure during study (mm Hg)	122 (22.1)	136.4 (28.5)	**0.007**
Diastolic blood pressure during study (mm Hg)	64 (16.1)	70.3 (16.8)	**0.049**
Ejection fraction (%)	58.50 (15.33)	56.05 (16.06)	0.452
Estimated pulmonary artery systolic pressure (mm Hg)	42.69 (15.08)	41.96 (12.15)	0.827
Aortic valve peak velocity (m/s)	3.06 (0.71)	3.26 (0.89)	0.253
Aortic valve mean gradient (mm Hg)	22.35 (10.73)	25.23 (14.21)	0.271
Aortic valve area (cm^2^)	1.32 (0.37)	1.05 (0.35)	**0.001**
Stroke volume (mL/beat)	84.38 (17.36)	66.06 (16.06)	**<0.001**
Stroke volume index (mL/m^2^/beat)	45.51 (8.87)	33.20 (8.90)	**<0.001**
Dimensionless index	0.40 (0.11)	0.33 (0.11)	**0.001**
Cardiac output (L/min)	6.16 (1.34)	5.30 (1.55)	**0.005**
Valvuloarterial impedance (Zva) (mm Hg/mL/m^2^)	3.22 (0.49)	4.99 (0.82)	**<0.001**
Outcomes			
Aortic valve replacement (AVR)	8 (17.4)	7 (14.6)	0.93
Mortality	24 (52.2)	18 (37.5)	0.22
Mortality and AVR	32 (70)	25 (52)	0.18

Values are n (%) unless otherwise indicated. **Bolded***P*-values indicate the *P*-value is significant (ie, <0.05).

## Discussion

Patients with ESKD experience a higher risk of incident AS; and higher risk of mortality in the context of coprevalent AS.^4^ In this high-risk population, accurate assessment of AS severity using echocardiography presents notable challenges.^4^ The use of the continuity equation for calculating AVA requires an accurate assessment of the left ventricular outflow tract diameter, which is problematic in ESKD due to prominent septal hypertrophy and extensive aortic annular/adjacent calcification owing to the unique metabolic milieu of secondary hyperparathyroidism.^4^ Furthermore, high flow states due to the presence of arteriovenous fistula and low flow states due to low CO have been described, impacting accurate calculation of AVA using the continuity equation.^3^^,^^4^ Finally, hypertension and reduced aortic compliance are common in ESKD but routine echocardiographic assessment does not factor in the contribution of the true global hemodynamic afterload experienced by the LV in the setting of AS.^3^

Our study attempted to determine the impact of global hemodynamic load; it is the first to evaluate correlates of Zva in patients with ESKD in a diverse safety-net population. Contrary to the findings of Hachicha et al, our study suggests that Zva does not predict the composite outcome of mortality or AVR in patients with ESKD.^1^ It is noteworthy that Hachicha et al studied a larger cohort (n = 544) with at least moderate AS.^1^ Also, Nuis et al studied a cohort of 250 patients undergoing transcatheter AVR; among 192 patients who survived >1 year following transcatheter AVR, elevated Zva correlated with quality of life/exercise performance.^2^ Levy et al^5^ studied Zva among 184 patients with severe, low ejection fraction, low-gradient AS; and similar to our study, found that while Zva correlated with echocardiographic markers (eg, low LV ejection fraction and contractile function), it failed to predict operative and long-term mortality.

Several explanations can be hypothesized to reconcile the observations from these studies. The larger cohort sizes in prior studies and their focus on patients with more advanced AS offer a likely explanation for differences from our study. Alternatively, our findings could reflect the heightened baseline risks in ESKD, blunting any additional prognostic impact of Zva in a high-risk population, similar to the findings by Levy et al. Moreover, our findings could be influenced by higher prevalence of hypertension in ESKD as well as differential flow states depending on type of dialysis access.^3^ Yet, the correlation between elevated levels of Zva (>4 mm Hg/mL/m^2^) with markers of severe AS (AVA) and low-flow state (CO/SVi) in our study highlight the echocardiographic variables relevant for composite clinical assessment of AS in ESKD.

This study is limited by the observational design, whereby we cannot infer causality or rule out unmeasured confounders. Due to the small size of the cohort, the possibility of type II error exists. Finally, as outlined above, this study was not limited to those with more advanced AS, in whom the role of Zva might be most prominent. Yet, to the best our knowledge, this is the largest cohort of coprevalent AS and ESKD, with patient-level echocardiographic data.

In conclusion, our findings suggest that Zva is associated with echocardiographic markers of severity of AS and low-flow state but not with clinical outcomes among patients with coprevalent ESKD and AS. Future studies should validate these observations in a larger cohort, ideally among patients with severe AS, where the prognostic role of Zva could be most clinically pertinent. We hope that our study inspires more studies in this vulnerable high-risk population of ESKD patients who have been historically excluded from most studies.

## Funding support and author disclosures

The authors have reported that they have no relationships relevant to the contents of this paper to disclose.
